# Establishing a rabbit model with massive supraspinatus tendon defect for investigating scaffold-assisted tendon repair

**DOI:** 10.1186/s12575-024-00256-z

**Published:** 2024-10-04

**Authors:** Shuting Huang, Ming Yik Tam, Wai Hon Caleb Ho, Hong Ki Wong, Meng Zhou, Chun Zeng, Denghui Xie, Dai Fei Elmer Ker, Samuel KK. Ling, Rocky S. Tuan, Dan Michelle Wang

**Affiliations:** 1 School of Biomedical Sciences, The Chinese University of Hong Kong, Hong Kong SAR, China; 2 Institute for Tissue Engineering and Regenerative Medicine, The Chinese University of Hong Kong, Hong Kong SAR, China; 3 Center for Neuromusculoskeletal Restorative Medicine, Hong Kong Science Park, Hong Kong SAR, China; 4 Department of Orthopaedics and Traumatology, Faculty of Medicine, The Chinese University of Hong Kong, Hong Kong SAR, China; 5 Department of Orthopedic Surgery, Center for Orthopedic Surgery, Guangdong Provincial Key Laboratory of Bone and Joint Degeneration Diseases, The Third Affiliated Hospital of Southern Medical University, Guangzhou, China; 6 Department of Biomedical Engineering, Faculty of Engineering, The Hong Kong Polytechnic University, Hong Kong SAR, China

**Keywords:** Massive rotator cuff tendon defect, Animal model, Tendon scaffold, Tendon tissue engineering

## Abstract

**Background:**

Shoulder pain and disability from rotator cuff tears remain challenging clinical problem despite advancements in surgical techniques and materials. To advance our understanding of injury progression and develop effective therapeutics using tissue engineering and regenerative medicine approaches, it is crucial to develop and utilize animal models that closely resemble the anatomy and display the pathophysiology of the human rotator cuff. Among various animal models, the rabbit shoulder defect model is particularly favored due to its similarity to human rotator cuff pathology. However, a standardized protocol for creating a massive rotator cuff defect in the rabbits is not well defined. Therefore, the objective of our study was to establish a robust and reproducible model of a rotator cuff defect to evaluate the regenerative efficacy of scaffolds.

**Results:**

In our study, we successfully developed a rabbit model with a massive supraspinatus tendon defect that closely resembles the common rotator cuff injuries observed in humans. This defect involved a complete transection of the tendon, spanning 10 mm in length and encompassing its full thickness and width. To ensure stable scaffolding, we employed an innovative bridging suture technique that utilized a modified Mason-Allen suture as a structural support. Moreover, to assess the therapeutic effectiveness of the model, we utilized different scaffolds, including a bovine tendon extracellular matrix (ECM) scaffold and a commercial acellular dermal matrix (ADM) scaffold. Throughout the observation period, no scaffold damage was observed. Notably, comprehensive histological analysis demonstrated that the regenerative tissue in the tendon ECM scaffold group exhibited an organized and aligned fiber structure, indicating tendon-like tissue regeneration while the tissue in the ADM group showed comparatively less organization.

**Conclusions:**

This study presents a comprehensive description of the implemented procedures for the development of a highly reproducible animal model that induces massive segmental defects in rotator cuff tendons. This protocol can be universally implemented with alternative scaffolds to investigate extensive tendon defects and evaluate the efficacy of regenerative treatments. The application of our animal model offers a standardized and reproducible platform, enabling researchers to systematically evaluate, compare, and optimize scaffold designs. This approach holds significant importance in advancing the development of tissue engineering strategies for effectively repairing extensive tendon defects.

## Background

 Rotator cuff tendinopathy and tears are a significant cause of shoulder pain and disability. Among these tears, the supraspinatus tendon is particularly susceptible, with a prevalence of 61.9% in men and 38.1% in women [1]. Massive rotator cuff tears involving multiple tendons, full-thickness tears larger than 5 cm, or significant tendon retraction [2], represent a substantial proportion of all rotator cuff injuries (40%) and recurrent tears (80%) [3]. These extensive injuries often result in reduced strength, limited range of motion (ROM), and debilitating pain. Generally, treatment strategies involve surgical repair for younger physically active patients and older patients who do not respond to conservative treatment [4]. However, despite advances in surgical techniques and materials, retear rates remain high, ranging from 40 to 90% [5]. There is thus a need to improve biological augmentation strategies to enhance tendon healing and minimize postoperative degeneration.

With advancements in tissue engineering, tissue scaffolds have emerged as a promising approach to augment rotator cuff repair by providing both mechanical support and favorable biological properties for tendon healing [4]. To evaluate the efficacy of these scaffolds and facilitate translation to clinical applications, rigorous in vivo assessments in preclinical animal shoulder models that simulate human anatomy, physiology, and pathology are essential. Among the available animal models, the rabbit shoulder model is preferred due to its close resemblance to human rotator cuff pathology. Previous studies on rotator cuff tears in rabbits have indicated similar chronic changes to those observed in human patients, including muscle atrophy and fatty infiltration [6]. More importantly, the larger size of rabbit allows for more accurate and reproducible tendon defects compared to other commonly used animal models like rats and mice, and can be performed using standard surgical techniques and equipment [7].

Furthermore, the design of the rotator cuff defect model is important and involves several key considerations, including selection of appropriate animal model (e.g., tendon defect site and size) and the strategy for scaffold implantation (e.g., the suturing technique, scaffold implantation method). Currently, there is no established standardized protocol for producing a massive rotator cuff defect in the rabbit model. Past studies have employed different approaches regarding tendon choice [6, 8–10], defect lengths [10–12], and locations along the tendon. Moreover, multiple techniques exist for scaffold implantation (e.g., augmentation and bridging) and suturing (e.g., Kessler’s, lock loop, Mason-Allen’s suture method) [13–15]. These variabilities pose challenges for comparative analysis. Hence, establishing a standardized model would enable more controlled evaluation of scaffold-mediated repair.

Our objective is to develop a robust and reproducible model of a rotator cuff defect for evaluating the regenerative efficacy of scaffolds. Nonetheless, the choice of implantation technique is influenced by the properties of the scaffold itself. Thus, when using a bridging scaffold that is connected in series with the tendon, it is essential for the scaffold to transmit all pulling forces across the bridged tendon ends during muscle activity [16]. Consequently, the scaffold needs to possess high mechanical strength. Considering the severity of tear, loss of tendon length, and the properties of the scaffold, proper bridging technique is critical for implantation. Additionally, proper sutures play a vital role in enabling appropriate force transmission, withstanding the pulling forces generated by muscle contraction, and preventing scaffold dislocation and gap formation under loading [17, 18]. In cases of simple tendon repair involving the tendon-tendon interface, gaps larger than 3 mm can lead to unfavorable clinical outcomes in dogs [19]. However, sutures can potentially interfere with the microcirculations in tendons [17, 20], hindering the blood supply necessary for the formation of fibrovascular tissues and subsequent regeneration [21]. Therefore, it is important to develop an optimal suture strategy that achieves scaffold stabilization while minimizing disruption to native tendon healing.

This study aimed to develop a robust model of a massive supraspinatus tendon defect model in rabbits. The model involved a complete transection of the tendon, spanning 10 mm in length and encompassing the full thickness and width of the tendon. To repair the tendon, we implemented both direct suture repair and scaffold-mediated repair utilizing two distinct scaffolds: the acellular dermal matrix (ADM, a commercially available scaffold for tendon repair) [22] and the tendon extracellular matrix (ECM) scaffold (a polyurethane scaffold enriched with tendon extracellular matrix) [23]. To ensure optimal stability of the scaffold, we employed an innovative bridging suture technique that utilized a modified Mason-Allen suture as a structural support. Tendon healing outcomes were assessed through histological analyses, including Hematoxylin and Eosin (H&E) staining, Picrosirius red staining, and histological evaluation scores.

## Materials and methods

### Animals

Twelve New Zealand White rabbits (13–16 weeks old) with an average body weight of 4 kg were obtained from the Laboratory Animal Services Centre, the Chinese University of Hong Kong. All procedures, including rabbit surgeries, were conducted using sterile surgical technique in accordance with protocol (No. 18-003-MIS) approved by The Chinese University of Hong Kong Animal Experimentation Ethics Committee in an appropriately equipped room designated for animal surgeries.

### Preparation of animals for rotator cuff surgery

1. Surgical instruments were autoclave-sterilized, and sterile gloves were worn throughout the procedure, which was carried out in a sterile operating field.
2. Anesthesia was induced in both female and male New Zealand White Rabbits by administering an intramuscular injection of a ketamine (35 mg/kg; Alfasan) and xylazine (5 mg/kg; Alfasan) mixture.
3. To maintain anesthesia in rabbits during the surgery, a 5 ml syringe containing the ketamine (35 mg/kg) and xylazine (5 mg/kg) mixture was prepared. The rabbit’s ear vein was located, and an indwelling needle was carefully inserted and connected to the syringe containing the anesthetic. The syringe was secured with gauze and medical tape.
4. The anesthetized rabbit was gently positioned in a supine position, ensuring that the surgical area faced upwards. The surgical area was shaved and cleansed by applying three alternating applications of betadine and 70% ethanol, applied in circular motions, starting from the inside and moving outward, using a cotton swab. Surgical drapes were placed to create a surgical window and maintain a sterile environment.

### Isolation of the supraspinatus tendon

1. A 3 cm incision was made in the shoulder to expose the rotator cuff tendons. The deltoid muscle was split, and skin and soft tissue retractors were used to create a surgical window, providing a clear view of the rotator cuff tissues. Acromioplasty was performed using a No.15 scalpel (Mingyue). Hemostasis was achieved, and the wound was irrigated with saline solution.
2. The supraspinatus tendon was identified and marked using a 3 − 0 suture (Arthrex FiberWire^®^). The supraspinatus tendon was sutured using a lock-loop suturing technique before detaching it.
3. A full-thickness defect, measuring approximately 10 × 5 mm, was created in the mid-substance of the supraspinatus tendon using a No.15 scalpel. The distance between the defect and the first lock-loop suture site was approximately 1–2 mm. The opposite end of the supraspinatus tendon was sutured using the lock-loop suturing method.

### Implantation of the scaffold

1. To achieve a secure attachment between the scaffold and the tendon, a modified Mason-Allen suture technique was utilized. After completing the lock-loop suture, all subsequent sutures were positioned behind the lock-loop to minimize the risk of suture pull-out. Prior to the surgical procedure, three holes were created in the scaffold. The dimensions of the scaffold were designed to closely resemble the width and thickness of the native rabbit supraspinatus tendon.
2. The first stitch was passed through one-third of the tendon’s width and then secured with a knot, using its own suture as the initial fixation. The second stitch emerged behind the first stitch and connected to one of the scaffold’s holes, forming a stable “cross” structure between the first and second stitches.
3. After the suture was passed through the scaffold, it traversed the tendon from behind the lock-loop suture. At this stage, the Mason-Allen method was employed to tie a “cross” knot at two-thirds of the tendon’s length, while simultaneously connecting the tensioned suture to the second hole of the scaffold. Following the final Mason-Allen knot, an additional knot was tied using the suture itself at the opposing end of the tendon, to enhance fixation of the scaffold-tendon connection. The same suturing technique was employed to secure the scaffold and tendon together at the other end of the tendon.

### Wound closure and post-surgery sterilization

1. After the implantation, post-surgery analgesic (0.01-0.05 mg/kg Buprenorphine, Alfasan) was administered subcutaneously to ensure pain relief for the rabbits.
2. To prevent infection, an anti-infective (20 mg/kg Cefalexin, Alfasan) was administered.
3. A suture (3 − 0 PGA) was used to carefully reapproximate the deltoid muscle tissue layer by layer, and the skin was closed with 3 − 0 silk sutures.
4. To sterilize the wound and clean up any blood, betadine was applied in circular motions using a cotton swab, starting from the inside and moving outward.
5. The rabbits were allowed to recover on a heating pad and subsequently allowed cage activity in their cages without immobilization.

### Sample harvesting and analysis

1. At the designated time point of postoperative 1 month, euthanasia was performed on the rabbits using a lethal dose of sodium pentobarbital (Alfasan), administered at a dosage of 60 mg/kg. Euthanasia was carried out in strict adherence to institutional animal ethics guidelines to ensure humane treatment of the animals.
2. The harvested samples were immediately immersed in a 4% (w/v) solution of paraformaldehyde (PFA, Sigma) for 48-hour fixation. The tissue samples underwent graded ethanol dehydration and were subsequently embedded in paraffin blocks.
3. The embedded samples were sectioned at 7 μm thickness, deparaffinized and then stained with hematoxylin and eosin (H&E) and Picrosirius red.
4. The stained sections were examined using bright field optics to visualize H&E staining or polarized optics for Picrosirius staining using a Nikon Ni-U Eclipse Upright Microscope, and images captured using a digital camera [Nikon, DSFi3].
5. To quantify the differences among the groups, the H&E-stained sections were evaluated using a semi-quantitative histopathological scale according to a reported grading system [24]. Four parameters, i.e., cellularity, vascularization, feature of inflammation, and collagen alignment, were quantified using a 0–3 grading scale: 0 (normal), 1 (slightly abnormal), 2 (moderately abnormal), and 3 (maximally abnormal). The average scores were used for comparison.

### Statistical analysis

All data were expressed as mean ± standard deviation (SEM) and compared by the Kruskal-Wallis test followed by post hoc pair-wise comparison using the Mann-Whitney U test [25]. Statistically significant differences are indicated by asterisks (*, *p* ˂ 0.05; **, *p* ˂ 0.01; and ***, *p* ˂ 0.001).

## Results

### Establishing a rabbit model of massive rotator cuff tendon defect

**Fig. 1:**
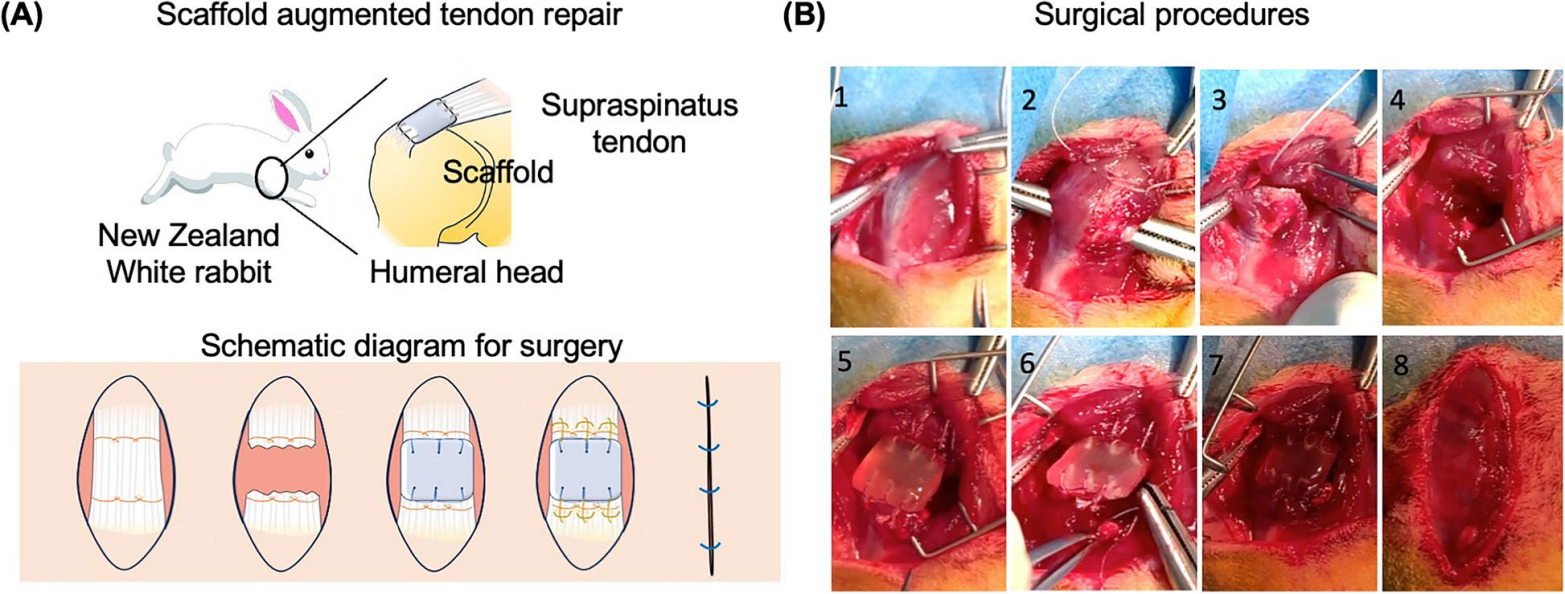
Schematic surgery diagram of repairing a massive rotator cuff tendon defect. **A** Diagram illustrating the creation of a massive rotator cuff tendon defects. **B** Representative images depicting surgical procedure steps

**Fig. 2:**
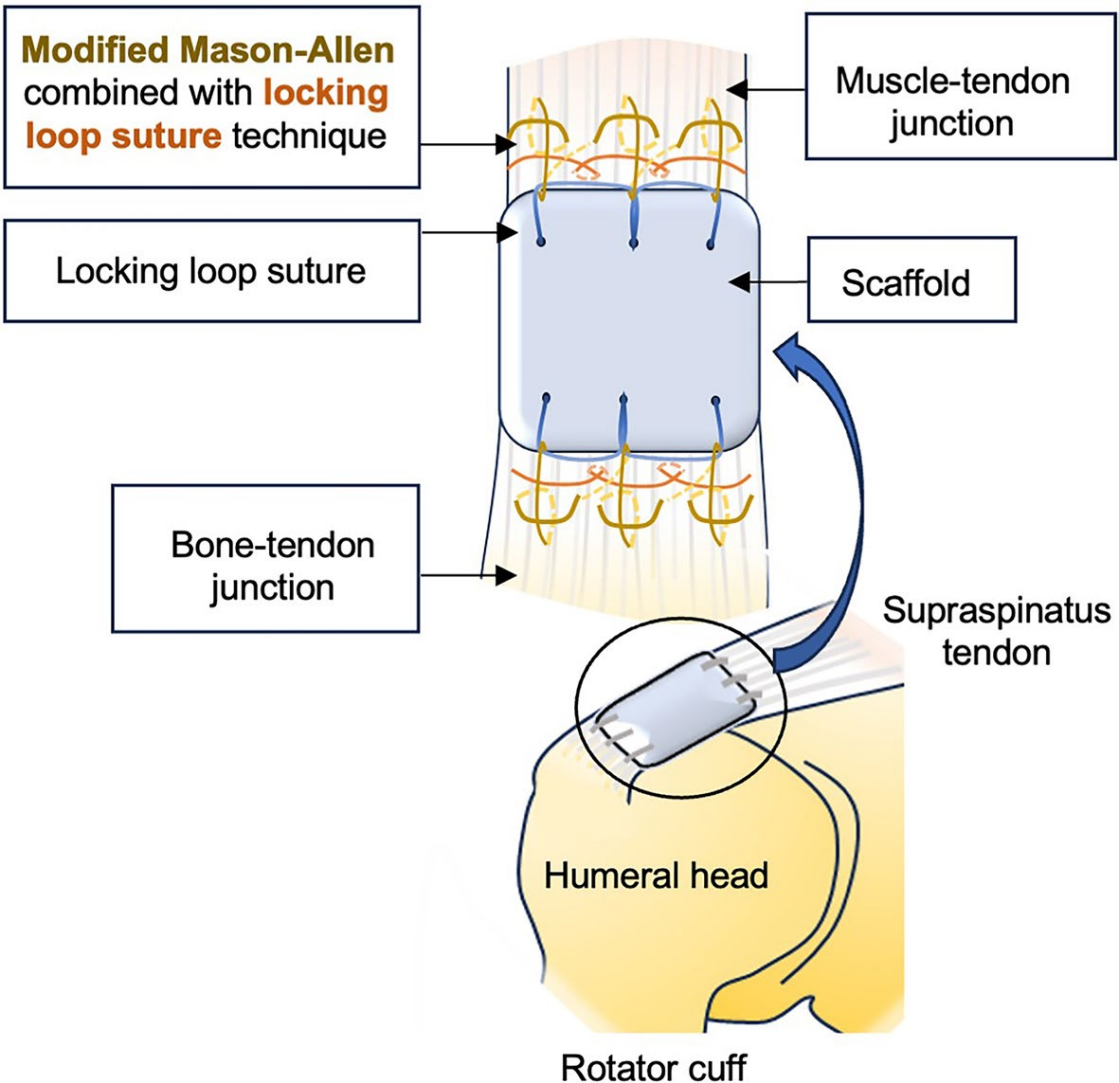
Schematic diagram illustrating the suture technique used for scaffold implantation. The highly stable implantation model was achieved by combining the modified Mason-Allen method with the locking loop suture method

To evaluate the tendon healing efficacy of different scaffolds (i.e., ADM and tendon ECM scaffold), a massive tendon defect model was created on the supraspinatus tendon of New Zealand White rabbits (Fig. 1A). As demonstrated in Fig. 1B, the supraspinatus tendon was identified following a 3 cm incision in the shoulder. The surrounding tissue was carefully isolated, and the supraspinatus tendon was sutured with a lock-loop suturing technique. The suture site was positioned 1 cm apart, and a full-thickness defect measuring 5 mm in length was created between the suture sites to simulate a significant tendon defect (Fig. 1B). To bridge the tendon defect using different materials, the scaffold was implanted between the defect site and connected to the two tendon ends via a modified Mason-Allen’s suture technique (Fig. 2). To ensure a tight connection between tendons and scaffold, the combination of lock-loop suture and Mason-Allen’s suture method was applied. This approach ensured a secure connection between the tendons and the scaffold. The modified Mason-Allen’s suture method was performed between tendon and scaffold, while the lock-loop suture method was employed on the tendon ends below the Mason-Allen’s suture knots. This design resulted in a tight and robust connection between the scaffold and tendon, significantly reducing the risk of suture pull-out.

### Assessment of tendon healing outcomes using histological analysis

**Fig. 3:**
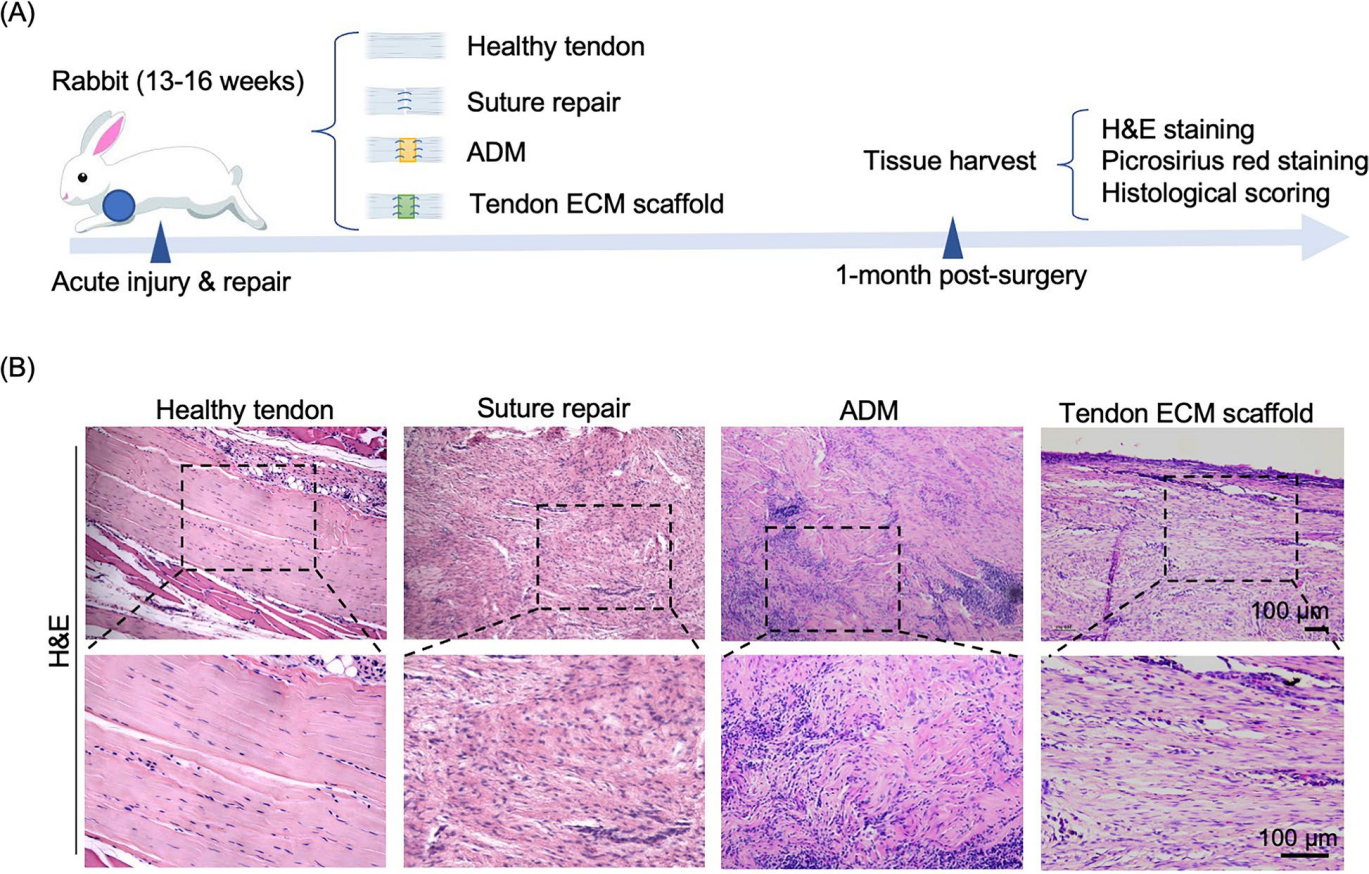
Histological analysis using H&E staining to assess healing outcome at 1 month after surgery. **A** Schematic diagram illustrating the experimental design. **B** H&E analysis revealing the evaluation of tendon healing outcomes. The tendon extracellular matrix (ECM) scaffold group demonstrated the presence of regenerative tissue with a well-aligned structure resembling that of a healthy tendon. In contrast, in both the suture repair and acellular dermal matrix (ADM)-mediated repair groups, a disorganized tissue structure with increased cellularity was observed, compared to the organized architecture seen in the healthy tendon group

To assess the efficacy of the designed scaffold (tendon ECM scaffold) in comparison to a commercially available scaffold (ADM), four experimental groups were established: a healthy tendon control group, a group undergoing direct suture repair, a group undergoing ADM-mediated repair, and a group undergoing tendon ECM scaffold-mediated repair (Fig. 3A). At 1 month, the rabbits were euthanized, and samples were collected for analyses, including H&E staining, Picrosirius red staining, and scoring analysis. Histologically, the H&E staining images demonstrated that both the ADM and tendon ECM scaffold groups displayed complete bridging of the 1-cm tendon defect and exhibited increased cellularity when compared to the intact control group. Additionally, in comparison to the suture repair and ADM-mediated repair groups, the regenerative tissue in the tendon ECM scaffold group exhibited a more aligned fiber structure, indicating a greater degree of tendon-like tissue regeneration.

**Fig. 4:**
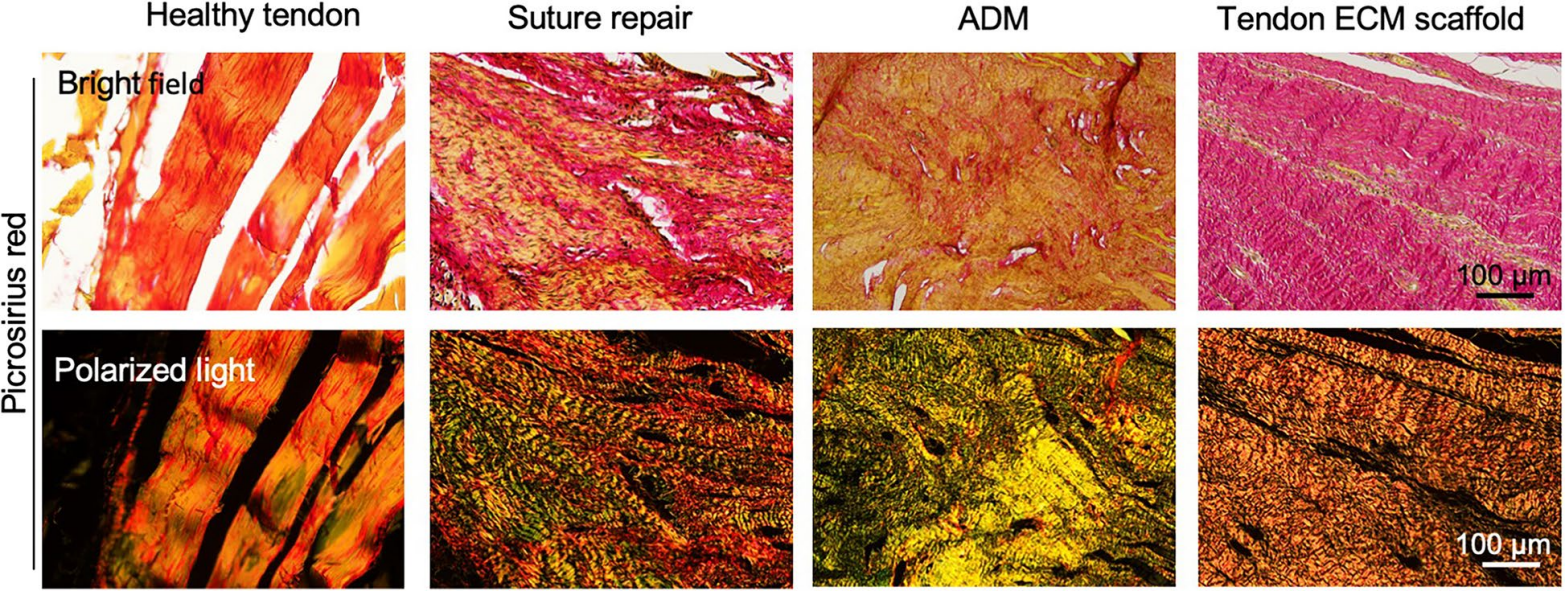
Histological analysis using picrosirius red staining and polarized light microscopy to assess the healing outcome at 1 month after surgery. In the tendon ECM scaffold group, a distinctly wavy and more aligned ECM structure was observed, characterized by a pronounced birefringence. Furthermore, a more abundant presence of thicker collagen fibers was observed compared to both the suture repair and ADM-mediated repair groups. However, the fiber thickness in the tendon ECM scaffold group remained lower than that observed in the healthy tendon group, suggesting an ongoing regenerative stage at the 1-month time point

To evaluate the alignment and thickness of collagen fibers, picrosirius red staining observed with polarized light microscopy was utilized. When viewed under polarized optics, the tendon ECM scaffold-mediated group exhibited a more pronounced birefringence compared to both the suture repair and ADM-mediated repair groups. Additionally, the tendon ECM scaffold group displayed a higher abundance of orange-to-red fibers, indicating thicker fibers, while the suture repair and ADM-mediated repair groups had a greater prevalence of yellow fibers, suggesting thinner fibers (Fig. 4). However, it is noteworthy that the fiber thickness in the tendon ECM scaffold-mediated groups remained lower than that observed in the healthy tendon group.

**Fig. 5:**
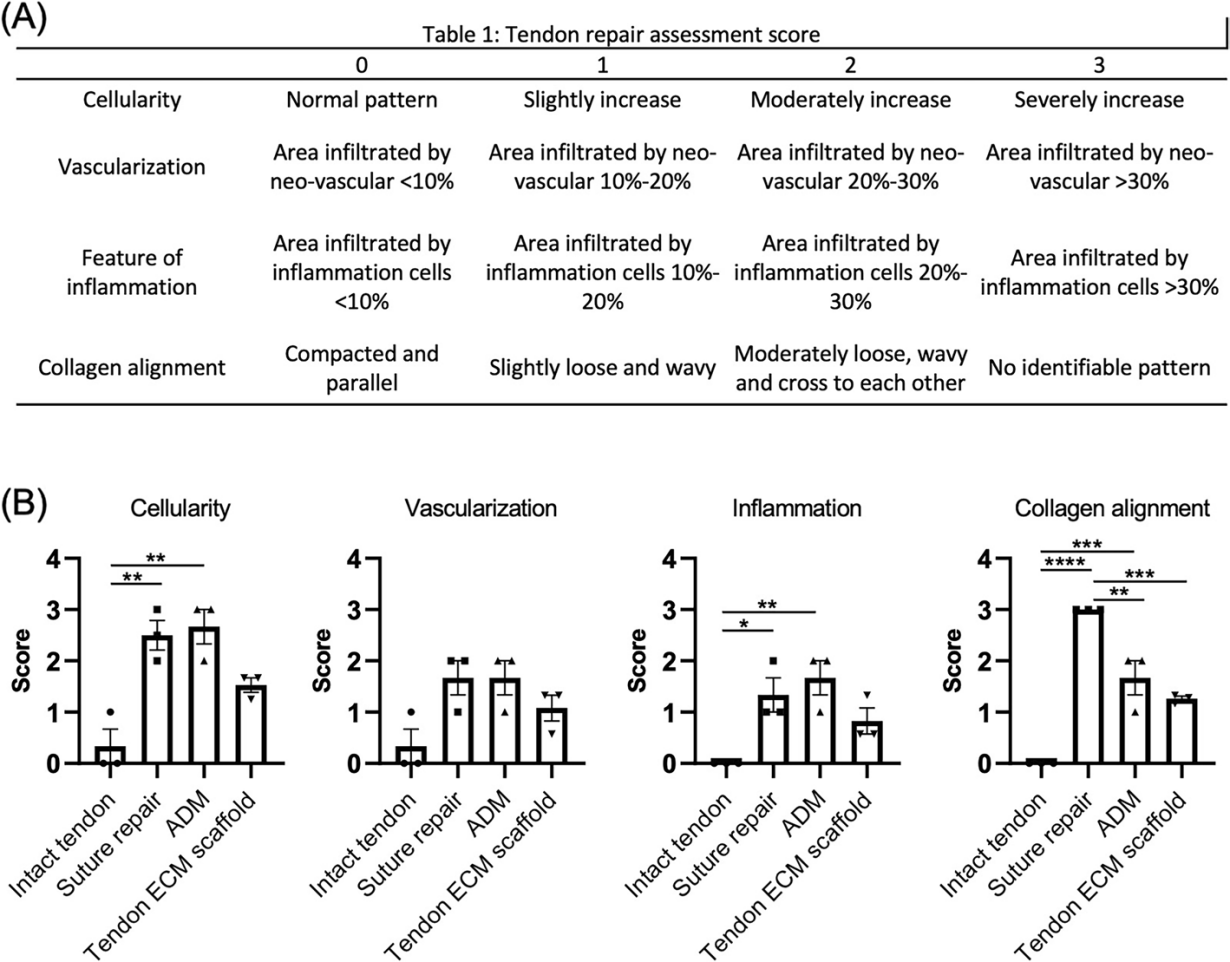
Histological evaluation and grading of tendon repair on the basis of Hematoxylin and Eosin (H&E) staining.** A **Grading system for tendon healing. **B **Scores assigned for cellularity, vascularization, inflammation, and collagen alignment. Both the suture repair and ADM-mediated repair groups displayed significantly higher cellularity and inflammatory response, as well as less organized collagen, compared to the healthy tendon group. In contrast, the tendon ECM scaffold group exhibited no significant differences compared to the healthy tendon group regarding cellularity, vascularization, and inflammation. *n* = 3, biological replicates; mean ± SEM; *, *p* < 0.05; **, *p* < 0.01; ***, *p* < 0.001; ****, *p* < 0.0001

Additionally, to evaluate tendon healing, a grading system [24] was implemented to assess the levels of cellularity, vascularization, inflammation, and collagen alignment in each group (Fig. 5A). The suture repair and ADM-mediated repair groups demonstrated significantly higher cellularity and inflammatory response, as well as less organized collagen, when compared to the healthy tendon group (Fig. 5B). In contrast, the tendon ECM scaffold group exhibited no significant differences in terms of cellularity, vascularization, and inflammation compared to the healthy tendon group (Fig. 5B). However, fiber alignment in the healthy tendon group remained significantly higher than that observed in all the repair groups, with the suture repair group displaying the least fiber alignment when compared to the ADM-mediated repair and tendon ECM scaffold repair groups (Fig. 5B).

These findings suggest that the implantation of the tendon ECM scaffold resulted in the formation of tendon-like tissue with improved organization and fiber thickness compared to the suture repair and ADM-mediated repair groups. Nonetheless, it is important to emphasize that despite the improved alignment and fiber thickness observed in the tendon ECM scaffold-mediated group, these parameters still fell short of reaching levels comparable to those observed in a healthy tendon. These findings highlight the ongoing regenerative process of the regenerated tendon tissue at the 1 month post-surgery time point.

## Discussion

 Rotator cuff injuries are a significant cause of pain, functional limitation, and morbidity [26]. The high prevalence of rotator cuff pathologies underscores the urgency for the development of innovative strategies or therapeutics to address these conditions [26]. Animal models have contributed greatly to the advancements in researching and alleviating the burden of rotator cuff injuries, owing to their capacity to replicate anatomical, biomechanical, cellular, and molecular aspects of the human rotator cuff [7]. Among animal models, rabbits are commonly utilized for studying rotator cuff tendon repair [7]. Compared to smaller animal models such as rats and mice, rabbits exhibit less spontaneous tendon healing following tendon injury [27]. Additionally, the supraspinatus muscle of rabbits’ experiences fatty degeneration after tendon detachment, which closely resembles the condition observed in humans [27]. The larger size of rabbits also allows for the ready use of surgical models and techniques, enhancing the accuracy and reproducibility of experimental procedures. Similar to larger animal models such as goats, sheep, and dogs, rabbits possess the advantages of accommodating standard-of-care surgical techniques, robust mechanical loading, and scaffold-based repair strategies [28]. Additionally, their more upright posture closely resembles human anatomy compared to larger animals, which often have limited overhead reaching abilities [28]. Moreover, rabbits offer a cost-effective option for research due to lower purchase and housing expenses [27]. To provide more specific details, we have summarized representative studies that utilized various animal models to investigate acute and chronic rotator cuff tears in Tables 1, 2, 3, 4, 5 and 6.

**Table 1:** Established mouse models of tendon defect

**Tendon(s)**	**Types of model**	**Defect procedure**	**Repair procedure**	**Timepoints**	**Objectives(s)**	**Comments**
Supraspinatus tendon	Acute	Unilateral sharp release	Not specified	Day 0, 7, 10, 14	Murine model of supraspinatus tendon repair [29].	**Advantages:** 1. High anatomic similarity to humans. 2. High physiological similarity to humans. 3. Amenable to genetic manipulation. 4. Cost-effective. 5. Easy logistic management. 6. Easy to attain large sample size. **Disadvantages:** 1. Small size complicates surgical procedures. 2. Robust spontaneous tendon healing potentially masks intervention efficacy.
	Chronic	Unilateral complete transection	Not specified	Week 1, 4	Gene expression patterns in the supraspinatus muscle after tear [30].	
Supraspinatus and infraspinatus tendon	Chronic	Unilateral complete transection + denervation	Not specified	Week 12	Mouse model of massive rotator cuff tears that is consistent with the pathophysiology of human [31].	
				Week 2, 6	The role of Trichostatin A in rotator cuff tear [32].	
		Unilateral detachment + denervation	Not specified	Week 1, 6, 12	Effects of knocking out poly(ADP-ribose) polymerase 1 [33].

**Table 2:** Established rat models of tendon defect

**Tendon(s)**	**Types of model**	**Defect procedure**	**Repair procedure**	**Timepoints**	**Objective(s)**	**Comments**
Supraspinatus tendon	Acute	Bilateral detachment	Mason-Allen-like technique	Week 1, 2, 4, 8	Effects of carrier vehicles (Paloxamer 407) on tendon healing [34].	**Advantages:** 1. High anatomic similarity to humans. 2. Cartilage degeneration post-injury mimics human pathophysiology. 3. Cost-effective. 4. Easy logistic management. 5. Easy to attain large sample size. **Disadvantages:** 1. Robust spontaneous tendon healing potentially masks intervention efficacy. 2. Certain anatomical structures differ from human counterparts. 3. Minimal muscular fat accumulation. 4. Small size complicates surgical procedures.
			Not specified	Week 2, 8, 16	Effects of post-operative activity level on healing response [35].	
				Week 4, 8	Effects of cannulated humeral implant to potentially deliver local bone marrow for improving healing [36].	
		Unilateral detachment	Mason-Allen technique	Week 2, 4	Effects of mesenchymal stem cells transduced with scleraxis for the regeneration of tendon–bone insertion site [37].	
				Week 1, 7	Effect of Achilles bursal tissue implants in tendon-to-bone healing [38].	
			Modified Mason-Allen technique	Week 2	Effects of antibiotic treatment with fluoroquinolone on tendon healing [39].	
		Unilateral transection	Modified Mason-Allen technique	Week 2, 8	Effects of aging on tendon-to-bone healing [40].	
				Day 5, week 4	Effects of rhPDGF on rotator cuff repair [41].	
		Bilateral excision	Mason-Allen technique	Week 3, 6, 12	Effects of cells from the tendon–bone interface to amplify healing [42].	
	Chronic	Bilateral detachment + 2, 4, 16 weeks delayed repair	Not specified	Week 4, 16 post-surgery	The role of increased repair tension on tendon-to-bone healing [43].	
		Unilateral detachment + 4 weeks delayed repair	Not specified	Week 4, 8 post-surgery	Effects of Rapamycin on tendon-to-bone healing in aging rats [44].	
		Unilateral detachment + denervation	Modified Mason-Allen technique	Week 10, 14, 18	Effects of adipose-derived cell supplementation on tendon-to-bone healing [45].	
		Unilateral transection + tendon end braiding	Mattress technique	Week 2, 4, 8, 16	Evaluation of the histologic healing process and mechanical characteristics of the interface between a fascia lata autograft and supraspinatus muscle [46].	
		Persistent impingement by shuttled implant	Not specified	Week 2, 4, 8	Establishing novel rat models of chronic rotator cuff injuries with a persistent impingement [47].	
Supraspinatus and infraspinatus tendon	Acute	Unilateral transection	Modified Kessler-loop lock technique	Week 4, 8	Effects of a synthetic graft with multilayered co-electrospinning nano-scaffolds for bridging rotator cuff tear [48].	
	Chronic	Unilateral transection + silicone implants	Not specified	Week 8, 13, 20	Evaluation of the capability of ultrashort echo time magnetization transfer MRI for the differentiation of injuries and the detection of fibrosis [49].	
		Complete transection + denervation	Not specified	Week 2	Akt/mTOR activity in muscle atrophy after rotator cuff tears [50].	
		Unilateral detachment + 16 weeks delayed repair	Mason-Allen technique	Week 24, 32	Efficacy of a novel electroconductive matrix to treat muscle atrophy and fat accumulation [39].	
Infraspinatus tendon	Chronic	Bilateral detachment + 12 weeks delayed repair	Not specified	Week 2, 4, 8 post-surgery	Effects and mechanism of healthy tendon stem cell-derived exosomes on tendon-to-bone healing in aged rats [51].	
		Complete transection + 12 weeks delayed repair	Simple interrupted suture technique	Week 2, 4, 8 post-surgery	Efficacy of healthy tendon stem cell-derived exosomes on promoting tendon-to-bone healing in aged chronic rotator cuff tears [52].

**Table 3:** Established rabbit models of tendon defect

**Tendon(s)**	**Types of model**	**Defect procedure**	**Repair procedure**	**Timepoint**	**Objective(s)**	**Comments**
Subscapularis tendon-bone complex	Acute	Bilateral dissection	Not specified	Week 2	Rabbit subscapularis muscle model for the study of rotator cuff lesions [9].	**Advantages:** 1. Adequately models muscle atrophy, fatty accumulation, and other degenerative changes. 2. Larger size facilitates surgical procedures. 3. Mild-tempered and easy to handle. **Disadvantages:** 1. Suboptimal cost-effectiveness. 2. Heightened susceptibility to stress-induced injury and mortality, particularly during surgical procedures, postoperative care, and environmental changes.
Infraspinatus tendon	Acute	Bilateral detachment	Modified Mason-Allen technique	Week 2, 4, 8	Tendon-bone healing effects of multilayer decellularized tendon slices graft [53].	
Supraspinatus and subscapularis tendons	Chronic	Bilateral detachment + 8 weeks delayed repair	Mattress technique	Week 8 post-surgery	Effects of two types of upper joint capsule reconstruction grafts [54].	
Supraspinatus tendon	Acute	Bilateral transection	Not specified	Week 4, 8, 12	Tendon-bone healing effects of preservation of native enthesis [55].	
		Bilateral incision	Not specified	Week 12	Effects of nano-calcium silicate mineralized fish scale scaffolds [56].	
		Bilateral detachment	Not specified	Week 6	The tendon-to-bone healing effects of platelet-rich plasma and ozone therapy [57].	
	Chronic	Bilateral detachment + 6 weeks delayed repair + Penrose drain	Not specified	Week 12	Histological and biomechanical changes in a rabbit model of chronic rotator cuff tears repaired by human dermal fibroblasts [58].	
				Week 18	Effects of adipose stem cell-derived exosomes [59].	
		Unilateral transection + dissection of soft-tissue + Penrose drain	Infinity stitch Modified Kessler technique	Week 8, 16	Architectural and physiological analysis of chronic tear and repair compared with age-matched control rabbit supraspinatus muscles [60].	
			Not specified	Week 1, 2, 4, 8, 16	The progression of muscle loss and fat accumulation of rotator cuff tear [61].	
		Bilateral tenotomy + 6 weeks delayed repair + Penrose drain	Simple stitches	Week 12, 18	The tendon-bone healing effects of microfracture apertures [62].	
		Full-thickness detachment + tendon end braiding + 8 weeks delayed repair	Not specified	Week 1, 3, 6, 9, 12 post-surgery	In vivo biomechanical and histological processes of the rerouting biceps tendon to treat chronic irreparable rotator cuff tears [63].

**Table 4:** Established canine models of tendon defect

**Tendon(s)**	**Types of model**	**Defect procedure**	**Repair procedure**	**Timepoints**	**Objective(s)**	**Comments**
Infraspinatus tendon	Acute	Unilateral detachment	Modified Mason-Allen technique	Week 0, 6 3, 6 months	Effect of using human acellular dermal matrix grafting [64].	**Advantages:** 1. Adequately models muscle atrophy, fatty accumulation, and other degenerative changes. 2. Tolerates various postoperative rehabilitation programs. 3. Produce joint loads to the rotator cuff that are comparable in magnitude to those experienced by humans. 4. Larger size facilitates surgical procedures. **Disadvantages:** 1. Low anatomical similarity to humans. 2. Intrinsic physiological changes introduce confounding variables. 3. Logistically and economically challenging to carry out large-scale studies.
			Simple sutures Modified Mason-Allen technique Krakow technique	Week 1, 2, 3, 6, 12	Applicability of the canine rotator cuff acute full-thickness injury model [65].	
		Unilateral full-thickness detachment	Mason-Allen technique	Week 6	Engineered tendon-fibrocartilage-bone composite (TFBC) and mesenchymal stem cell sheet for augmentation using NWB model [66].	
		Bilateral sharp detachment	Mason-Allen technique	Week 12	Augmentation with a newly designed poly-L-lactide repair device [67].	
		Sharp transection	Not specified	Week 6	Non-weight-bearing model with radial neurectomy for research [68].	
	Chronic	Unilateral detachment + PRECLUDE wrap	Not specified	Week 12	Chronic injury model to explore the dynamic performance, muscle volume, and fat infiltration of infraspinatus muscles [69].	
		Unilateral transection + 3 weeks delayed repair	Suture-bridge technique	Week 0, 4, 8, 12, 24	Comparison of a bone-tendon allograft technique with a human dermis derived patch for reconstructing chronic large rotator cuff defects [70].	
		Bilateral transection + 4 weeks delayed repair	Mattress technique Suture-bridge technique Simple interrupted suture technique	Week 12	Effects of the interposition of demineralized cancellous bone matrix sponge hydrated in platelet-rich plasma on tendon-to-bone healing [71].	
Supraspinatus tendon	Acute	Bilateral half-thickness resection	Simple suture	3, 6 months	Augmentation of partial rotator cuff tears of different biologic scaffolds [72].

**Table 5:** Established ovine models of tendon defect

**Tendon(s)**	**Types of model**	**Defect procedure**	**Repair procedure**	**Timepoints**	**Objective(s)**	**Comments**
Infraspinatus tendon	Acute	Unilateral detachment	*Single-row:* Arthroscopic Mason-Allen stitches *Double-row:* Arthroscopic Mason-Allen stitches (lateral of the tendon) Mattress stitches (medial of the tendon)	Week 6, 12, 26	MRI–derived morphologic changes between single- and double-row rotator cuff repair and biomechanical properties [73].	**Advantages:** 1. High anatomic similarity to humans. 2. Good availability. 3. Cost-effective. 4. High societal acceptance as a research model. 5. Suitable for acute studies. 6. Larger size facilitates surgical procedures. 7. Adequately models muscle atrophy, fatty accumulation, and other degenerative changes. **Disadvantages:** 1. Certain anatomical structures differ from human counterparts. 2. Prone to tendon-bone interface gaps and excessive scar tissue formation post-injury. 3. Difficult to control the locomotive activity of large animals. 4. Post-operative management is logistically and economically challenging.
			Modified Mason-Allen technique Suture-bridge technique	Week 12	Effects of a double-row and a single-row technique on tendon blood flow [74].	
			Modified Mason-Allen technique	Week 12	Effects of inter-positional graft consisting PDGF-BB & a type I collagen matrix on tendon repair [75].	
				Week 4, 8	Effects of rhBMP-12 on the healing of rotator cuff repairs [76].	
			Double-row technique	6 months	Effect on rotator cuff repair of engineered tissue grafting [77].	
		Unilateral transection	Mattress technique	6 months	Muscle atrophy, fatty infiltration and fibrosis after repair of acute rotator cuff injury [78].	
			Mattress-like technique	Week 12	Biomechanical and histological characteristics of autografting in reconstruction of an infraspinatus defect by using different fixations [79].	
			Modified double-row technique	Week 6, 12	Mechanical, structural, and histologic quality of rotator cuff repairs augmented with an interposition electrospun nanofiber scaffold [80].	
		Unilateral central defect	Not specified	Week 12	Effect on rotator cuff repair of perforated anchors, or collagen scaffolds loaded with tenocytes [81].	
		Sharp removal	*Single-row:* Arthroscopic Mason-Allen stitches *Double-row:* Arthroscopic Mason-Allen stitches (lateral of the tendon) Mattress stitches (medial of the tendon)	Week 1, 2, 3, 6, 12, 26	Expression of different collagen types between double-row and single-row rotator cuff repair [82].	
		Sharp detachment	Single-loop stitches Modified Mason-Allen technique	4, 24 hours	Potential of tendon collagen crosslinking on improving suture pullout [83].	
		Bilateral detachment	Modified Mason-Allen technique	Week 4, 8	Biomechanical evaluation of the relation between number of suture anchors and strength of the bone-tendon interface [84].	
	Chronic	Unilateral detachment + 6, 18 weeks delayed repair + PRECLUDE wrap	Modified Mason-Allen technique	Week 12, 20, 30	Construction of chronic rotator cuff injury repair model [85].	
		Unilateral detachment + 4 weeks delayed repair + silicone implants	Modified Mason-Allen technique	Week 12	Effects of novel combination growth factor treatment incorporated into a PVA-Tyr hydrogel on enthesis healing [86].	
			Mason-Allen technique Mattress technique	3 months	Evaluation of 2 repair techniques with respect to biomechanical function [87].	
		Unilateral detachment + 6 weeks delayed repair + silicone implants	Suture-bridge technique Mason-Allen technique	Week 6, 12 post-surgery	Feasibility of using chitosan-platelet-rich plasma implants in conjunction with suture anchors to treat rotator cuff tears [88].	
		Unilateral osteotomy + silicone implants	Not specified	Week 14	Histological changes induced by anabolic steroids or IGF in experimentally degenerating rotator cuff tendons [89].	
			2 figure-of-8 stitches	Week 16	Quantification of the infraspinatus muscle work as the primary functional effect of chronic tendon tears on muscle [90].	
			Locked screw fixation technique Figure-of-8 stitches	Week 0, 16, 22, 34	Adipogenic and myogenic gene expression in infraspinatus muscle in a sheep animal model of chronic rotator cuff tears [91].	
				Week 0, 16, 22, 34	Reversion of structural muscle changes caused by chronic rotator cuff tears using continuous musculotendinous traction [92].	
		unilateral release + silicone implants + denervation	2 figure-of-8 stitches	Week 0, 6, 16	Mechanisms of muscle atrophy and degeneration after rotator cuff injury [93].	
		Unilateral release with bone chip + silicone implants + denervation	Not specified	Week 8, 16	Tenotomy predominantly induces fatty infiltration, and denervation induces mostly muscle atrophy [94].	
		Unilateral detachment + 40 weeks delayed repair + silicone implants	Not specified	Week 0, 16, 40, 42, 46, 52, 75	Associated muscular changes that occur with chronic rotator cuff tears [95].

**Table 6:** Established primate models of tendon defect

**Tendon(s)**	**Types of model**	**Defect procedure**	**Repair procedure**	**Timepoints**	**Objective(s)**	**Comments**
Supraspinatus tendon	Acute	Unilateral complete division	Not specified	Week 4, 8, 12, 16	Establishment of a model for supraspinatus tendon repair in baboons [96].	**Advantages:** 1. High anatomical similarity to humans. 2. High physiological similarity to humans. 3. Produce joint loads to the rotator cuff that are comparable in magnitude to those experienced by humans. **Disadvantages:** 1. High cost. 2. Complexity of management. 3. Challenging to justify ethical approval.
		Unilateral excision	Not specified	3, 6 months	The repair effect and immune response of a non-crosslinked porcine dermal extracellular matrix graft [97].	

To replicate massive rotator cuff tears observed in humans, a 10 mm segment of the rabbit supraspinatus tendon was used in this study, representing approximately 50% of the total tendon length [98]. The purpose was to simulate tendon retraction and atrophy following a supraspinatus tear [98]. Similar approaches have been employed in studies conducted by Yokoya et al. and Zheng et al. [10, 12]. Different repair strategies have been employed. In some studies, the torn tendon was directly pulled back into its original position before being anchored with sutures, resulting in excessive tension on the repaired tendon [99]. This increased traction force during augmentation can weaken the initial fixation and increase the risk of retear [100]. Conversely, other studies completely transected the tendon without removing any tendon tissue and directly bridged it with a scaffold, leading to a postoperative tendon length that exceeds 150% of the normal length [101, 102]. However, this method may result in insufficient mechanical stimulation within the defective tendon, ultimately leading to poor functional recovery. In our study, we utilized scaffolds specifically designed to match the dimensions of the 10 mm defect and bridge the tendon stumps. This design allowed us to achieve a tension level that closely mimics a normal tendon in the repair construct. By employing this approach, our aim was to enhance the accuracy and effectiveness of our experimental model.

An essential aspect that influences the success of tendon repair is the choice of a suitable suture strategy, which is crucial in ensuring the stability of the scaffold and preventing suture rupture or the formation of gaps under significant mechanical stress [103]. In our study, we primarily employed the modified Mason-Allen suture method, known for its high load-to-failure and superior resistance to gap formation [104]. However, given the specific conditions of our repair site, the modified Mason-Allen suture alone was deemed insufficient. Previous literature has introduced the concept of incorporating a row of medial fixation that runs perpendicular to the tendon fibers, serving as a rip-stop structure [105, 106]. Consequently, we opted to utilize the Ford interlocking suture technique to establish a rip-stop barrier. This approach reinforced the modified Mason-Allen suture and further minimized the potential for gap formation. By combining these two suturing approaches, we aimed to provide immediate mechanical support to the defective tendon until functional recovery occurs. It is worth noting that our strategy may result in an increased surface area of suture exposure on the tendon, which could potentially promote adhesion formation. However, this drawback can be overcome by implementing passive or active mobilization of the joint and tendon during the rehabilitative phase [17]. By doing so, we can effectively counterbalance the potential disadvantages associated with the use of additional rows of interlocking stitches. Ensuring the reproducibility of results in experimental animal research faces challenges [107]. The issue of poor reproducibility in animal research primarily arises from biological variation and experimental design [107].

To address the challenge of biological variance, we selected rabbits as our animal model due to their resemblance to human rotator cuff pathology. Regarding experimental design variance, we implemented several measures. Firstly, we carefully selected rabbits within the age range of 13–16 weeks, with an average weight of 4 kg, to minimize the influence of age-related factors. This selection aimed to reduce variations associated with different developmental stages. While a large proportion of massive rotator cuff tears are reported in elderly patients, it is important to recognize that adolescents [108] and adults under 40 years old working as manual labourers or actively serving in the military [109] are also affected and experience challenges not typically observed in older patients. These include unsatisfactory rates of return to physical activity or work at preinjury levels [110]. When defining the biological parameters for our rabbit model, both age and body weight were used since innate physiological and biomechanical differences in humans and rabbits made considerations based on age alone challenging. Specifically, the age and body weight of rabbits used herein was chosen to strike an appropriate balance for modelling both young and adult patients as 91% of mature adult body length is achieved 16-weeks postnatally while our rabbits’ mean body weight was 4 kg and about 90% similar to 1.5- to 2.5-year old mature adult rabbits [111]. Furthermore, numerous studies involving rabbit rotator cuff animal models have been reported with similar age (12–16 weeks old) [112] and body weights (2 to 2.5 kg [113] or 3 to 4 kg [112, 114]). Secondly, we included both male and female rabbits in the study to account for potential sex-based differences. Additionally, we provided a clear and detailed step-by-step description, along with schematic illustrations, of the surgical procedure used to create a massive supraspinatus tendon defect in rabbits. By doing so, we aimed to minimize potential variations arising from diverse surgical techniques. By taking these measures to mitigate biological and experimental design variances, we aimed to enhance the reproducibility of our findings and promote consistency in future studies utilizing our model.

To assess the outcomes of tendon repair, we conducted histological analysis using H&E staining (bright field optics) and Picrosirius red staining (polarized optics). Additionally, we implemented a scoring system to evaluate various parameters, including cellularity, vascularization, inflammation, and collagen alignment. Our findings demonstrated that the group receiving scaffold implantation exhibited superior healing outcomes in terms of collagen alignment compared to the group undergoing suture repair alone. Furthermore, we observed that different scaffold types yielded distinct results. Specifically, the tendon ECM scaffold group showed improved outcomes in terms of vascularization, inflammation, and collagen alignment when compared to the ADM group. These results indicate that the healing effect is not solely dependent on the suture techniques employed but is also strongly influenced by the specific implanted scaffold utilized.

Our rabbit rotator cuff tear model more closely resembles clinical acute rotator cuff injuries, rather than chronic injuries, which are more clinically predominant [115]. Acute rotator cuff injuries are generally defined as those occurring within 2 weeks to 6 months, with a “traumatic” onset following a shoulder trauma [116]. These acute injuries are commonly encountered in clinical orthopedics, with a reported incidence of 8% [117] and a prevalence of up to 40% of all rotator cuff tears [118]. Moreover, acute tears are a common cause of morbidity in the elderly, with an estimated incidence of 2.5 per 10,000 patients aged 40–75 years [119]. Particularly, acute rotator cuff tears after shoulder dislocation are particularly common in older patients, with rates of 54% in those aged 40–87 years [120] and 49% in those aged 60–89 years [121]. However, traumatic rotator cuff tears can occur in patients of all ages and lead to short- and long-term disability if not appropriately managed [115]. A literature review found that 37.6% of rotator cuff tears were attributed to trauma, with the majority caused by simple falls [122]. For young and healthy adults, the forces during falls can be great enough to tear even a tendon without degenerative changes [122], while the risk is higher for the elderly due to their greater risk of falling and poor tendon quality [122, 123]. The commonly involved tendons in acute rotator cuff tears are supraspinatus (84%), infraspinatus (39%), and subscapularis (78%) [26]. Interestingly, a prospective study showed that 50% of patients initially diagnosed with full-thickness rotator cuff tears and receiving conservative treatment had enlargement of tear size after 1 year [124], suggesting the need for early surgical repair, which correlates with previous follow-up studies [125, 126]. Patients with symptomatic rotator cuff tears typically experience pain, weakness, loss of function, and cascading effects on sleep, work, leisure, and psychosocial functioning, including depression and anxiety [127], necessitating early surgical repair. In contrast, patients with chronic full and partial thickness tears due to tendon degeneration and attrition are not always referred to the hospital unless they have substantial problems [128]. Therefore, further study on acute rotator cuff tear models is needed before progressing to more complicated chronic rotator cuff tear models.

One major drawback of using animals to model human rotator cuff tears is that most animals rely on their limbs for support, and their forelimbs have more weight-bearing functions than humans [27]. Rabbits, rats, dogs, and sheep lack the blending of individual flat tendons to form a single insertion, which is a defining feature of the human rotator cuff anatomy [129]. However, animal models still serve as practical means to understand the cellular and molecular pathways and pathology of rotator cuff tears and to develop new technologies to improve existing treatments. Ideal animal models of rotator cuff repair should lack spontaneous tendon healing after injury, have tendon sizes that allow for suture repair techniques like those used in humans, and exhibit irreversible muscular atrophy, stiffness, and fatty accumulation after injury [27]. A systematic review found that the rat model is most used (53.56%), followed by the rabbit model (25.67%), with the supraspinatus tendon being the most common injury site (62.10%), and acute full-thickness tear being the most common injury type (48.41%) [130]. The most common research purposes were testing the repair effect of patches (24.94%), observing pathophysiological changes after rotator cuff injury (20.78%), and testing the intervention effect of drugs (11.00%).

## Conclusions

In summary, this study successfully developed a massive rotator cuff tendon defect model in rabbits. Furthermore, a scaffold-mediated approach utilizing a modified Mason-Allen suture technique was specially designed as the repair strategy. This technique ensured a secure and tight connection between the damaged tendon and the implanted scaffold. The defect model and repair strategy developed here represent a highly practical animal model for conducting a wide range of preclinical studies aimed at evaluating the efficacy of tissue engineering-based tendon repair methods. This comprehensive protocol provides a powerful tool for studying massive rotator cuff tendon defects and facilitates the development of novel, tissue engineering based therapeutic strategies for tendon repair.

## Data Availability

Data sharing is not applicable to this article as no datasets were generated or analyzed during the current study.

## Abbreviations

ADM: Acellular dermal matrix
ECM: Extracellular matrix
H&E: Hematoxylin and Eosin
PFA: Paraformaldehyde
ROM: Range of motion
SEM: Standard deviation

## Data availability

No datasets were generated or analysed during the current study.
